# Biomechanical tests and finite element analyses of pelvic stability using bilateral single iliac screws with different channels in lumbo-iliac fixation

**DOI:** 10.3389/fsurg.2022.1035614

**Published:** 2022-11-08

**Authors:** Yangyang Sun, Ying Fu, Fanxiao Liu, Wen Zhang, Huanzhi Ma, Qinghu Li, Dongsheng Zhou, Baisheng Fu

**Affiliations:** ^1^ Department of Orthopaedic Surgery, Qilu Hospital (Qingdao), Cheeloo College of Medicine, Shandong University, Qingdao, China; ^2^ Shandong Center for Disease Control and Prevention, Jinan, China; ^3^ Department of Orthopaedic Surgery, Shandong Provincial Hospital Affiliated to Shandong First Medical University, Jinan, China; ^4^ Institute of Orthopedics, Soochow University, Suzhou, China

**Keywords:** lumbo-iliac fixation, biomechanical tests, pelvic, finite element analyses, internal fixation

## Abstract

**Background:**

In lumbo-iliac fixation, the iliac screw can be placed in several locations and directions. There is no uniform standard for the placement of a single iliac screw. Biomechanical tests and finite element analyses were used to compare the effect of bilateral single iliac screws with three channels on pelvic stability to determine the best channel.

**Methods:**

Five embalmed adult cadaver pelvic specimens were selected. An unstable Tile C1 pelvic injury model was established. Lumbo-iliac fixation for the treatment of left sacral Denis II fracture includes the following: three channels of bilateral, single iliac screws (channel A from posterior superior iliac spine (PSIS) to anterior inferior iliac spine (AIIS), channel B from 1 cm medial and 1 cm caudal of PSIS to AIIS, and channel C from 2 cm below PSIS to AIIS). Biomechanical testing was performed for stiffness evaluations. A finite element model was established to study the stress distribution of the model and the maximum von Mises stress of internal fixation.

**Results:**

Biomechanical tests revealed that under vertical compression loading. The compressive stiffness fixed by channel B (246.15 ± 27.85 N/mm) was better than that fixed by channel A and channel C. Under torsional load, the torsional stiffness fixed by channel B (2.234 ± 0.223 N·m/°) was stronger than that fixed by channel A and channel C. However, there was no significant difference in terms of compressive and torsional stiffness between channel B and channel A (*P* > 0.05). Finite element analyses conformed that the maximum von Mises stress of the internal fixator fixed in channel B under the conditions of vertical, forwards bending, backwards extension, left bending, left rotating, and right bending (213.98 MPa, 338.96 MPa, 100.63 MPa, 297.06 MPa, 200.95 MPa and 284.75 MPa, respectively) was significantly lower than those fixed in channel A and channel C.

**Conclusions:**

The construct stiffness of the channel from 1 cm medial and 1 cm caudal of PSIS to AIIS is better than that of the other two channels. This channel has the advantages of good biomechanical stability, small maximum von Mises stress of internal fixation.

## Introduction

Sacral fractures often caused by high energy injuries, such as car accidents and falls, account for 17%–30% of pelvic fractures (1) and are commonly accompanied by fractures of other bones, nerve injury and internal organ injury (2). The main reason for disability and dysfunction in the later period of patient recovery is lumbosacral plexus injury (3). The treatment of spinal-pelvic separation caused by complex sacral fractures is complicated. Failure to perform adequate fixation will seriously affect the stability of the posterior pelvic ring and lumbosacral region. Iliolumbar fixation has become a reliable fixation method (4). Käch et al. (5) reported for the first time that five patients with unstable longitudinal vertical fractures of the sacrum (Denis II or III fractures) were treated with an L5 pedicle screw combined with iliac screw fixation. The follow-up results showed that the internal fixation was reliable. Schildhauer et al. (6) proposed a triangular fixation technique that combined a spinal-pelvis fixation system with sacroiliac screw fixation.

In lumbo-iliac fixation, the screw-rod connection system is very flexible. The iliac screw can be placed in many locations and directions, and multiple screws can be placed to enhance the fixation effect. There were multiple anchoring channels during iliac screw fixation: from PSIS to the iliac crest (7), from PSIS to AIIS (8, 9), from 1 cm medial and 1 cm caudal of PSIS to AIIS (10), from 2 cm below PSIS to AIIS (11), and from posterior inferior iliac spine (PIIS) to AIIS (12). At present, there is no uniform standard for the placement of iliac screws, nor has the biomechanical effect of bilateral single iliac screws with different channels on pelvic stability been explored.

According to a study of biomechanical conduction and mechanical distribution, the fixation strength of iliac screws in the lower column of iliac bone was higher than that in the upper column of iliac bone (13). The most commonly used channel in the clinic is from PSIS to AIIS. Many scholars believe this channel can insert into the iliac screw with the maximum length and diameter. Biomechanical studies mostly used this channel for experimental research (13, 14). However, the clinical application of iliac screws through this channel is prone to induce complications such as local skin necrosis caused by the protrusion of the implant, which may cause serious consequences, such as incision infection and the exposure of the implant (15). During the operation, part of the bone of the PSIS needs to be removed. Harrop et al. (10) proposed a modified iliac screw fixation technique that used PSIS 1 cm medial and 1 cm caudal to AIIS. It was pointed out that the modified channel was more convenient and practical in clinical application than the “traditional” channel and had fewer complications. It has been suggested that the advantages of this modified iliac screw channel are due to the proximity of the screws to the midline, eliminating the need for additional connectors and special bent rods during surgery, and the safety of the procedure due to its location on the medial side of the iliac crest, which eliminates the need to remove part of the posterior superior iliac spine bone (16). Schwend et al. (11) proposed a channel from 2 cm below PSIS to AIIS. Through autopsy and biomechanics, it was confirmed that the mechanical strength of the iliac screw in this channel was more than three times that of the traditional Galveston system. Tian et al. (12) found that the channel from PIIS to AIIS was below or just on the edge of the sciatic notch in approximately 61.1% of Asian pelvic specimens. There was the possibility of nerve injury. The authors pointed out that iliac screw placement in this channel was not recommended.

This study aimed to compare the effect of bilateral single iliac screws with three channels on pelvic stability in lumbo-iliac fixation using biomechanical tests and finite element analyses. First, a pelvic Tile C1 injury model was constructed: pubic symphysis separation and Denis II fracture of the left sacrum. Second, lumbo-iliac fixation for pelvic instability injuries and bilateral single iliac screws with three channels: channel A from PSIS to AIIS, channel B from 1 cm medial and 1 cm caudal of PSIS to AIIS, channel C from 2 cm below PSIS to AIIS. Third, biomechanical tests and finite element analyses were used to analyse the biomechanical mechanism and determine the best iliac screw placement channel to provide a scientific basis for practical and successful clinical application.

## Methods

### Preparation of pelvic specimens

**Figure 1:**
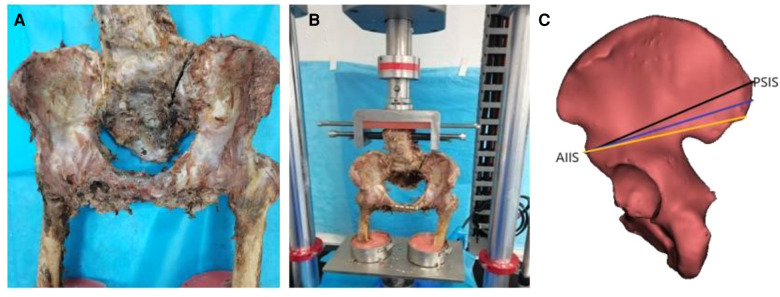
(**A**) An unstable tile C1 type injury (pubic symphysis separation and left sacral Denis II fracture) of pelvis specimen; (**B**) Frontal view of pelvic injury model (pubic symphysis fixed with a five-hole reconstruction plate and pelvic posterior ring injury treated by lumbo-iliac fixation) and biomechanical testing machine; (**C**) Three channels of single iliac screws.

The study protocol (2017GSF18112) was approved by the Ethics Committee of Shandong Provincial Hospital. Five adult embalmed cadaveric pelvic specimens (stored in a 10% formalin solution for two weeks and provided by the Department of Anatomy, Shandong First Medical University) were selected, including three males and two females, aged 42–58 years old, with an average age of 48.2 years old. The cadaver was separated at the L2–L3 joint and at 10 cm distal of the acetabulofemoral joint (Figure 1). Pelvic fracture, tumour, forced spondylitis, sacroiliac sclerosis, rheumatoid arthritis and other diseases were excluded by examination, and specimens proven to have osteoporosis using an osteocore 3 dual energy x-ray osteodensitometer (Medilink Company, Parc de la Mediterranee, France) were excluded (Table 1). Specimens were wrapped in double plastic bags and stored at −20°C. The specimens were thawed at room temperature. Skin, muscle, fat, and other tissues were removed. The complete pelvic bone, ligament structure, and hip joint were retained. The ligament structure mainly included the suprapubic ligament, pubic arch ligament, posterior sacroiliac ligament, anterior sacroiliac ligament, interosseous sacroiliac ligament, and hip joint accessory ligament. The upper and lower ends of the specimens were embedded with methyl methacrylate-polymer resin to enable fixation to the mechanical testing machine.

**Table 1:** General information on pelvic specimens and sequence of internal fixation.

Sequence number	Age (years)	Bone mineral density (*T* score)	Sequence of fixation
1	42	0.3	Channel A—channel B—channel C
2	50	0.2	Channel A—channel C—channel B
3	46	0.3	Channel B—channel C—channel A
4	58	0.2	Channel C—channel B—channel A
5	45	0.3	Channel B—channel A—channel C

### Establishment and fixation of the pelvic tile C1 injury model

After cadaver slippage was eliminated, experimental results from biomechanics testers of complete pelvis specimens were used as a control group. Then, the pubic symphysis was cut with an electric saw, and a left sacral Denis II fracture (sacral foramina fracture) (6) was established (Figure 1). Professor Baisheng Fu subsequently performed a series of surgical procedures. Anatomical reduction of the pelvic fracture was performed. A five-hole reconstruction plate was used to fix the separated pubic symphysis. The lumbo-iliac fixation was done for the treatment of unstable posterior pelvic ring injury. Lumbo-iliac fixation was completed with the L4 and L5 pedicle screws (6.5-mm diameter, 45-mm long) and iliac screws (7.5-mm diameter, 80-mm long), Medtronic-WeiGao Inc., WeiHai, China). The pedicle screws were laterally straight and parallel to the vertebral endplate. The bilateral single iliac screws were entering from three channels including channel A from PSIS to AIIS, channel B from 1 cm medial and 1 cm caudal of PSIS to AIIS, and channel C from 2 cm below PSIS to AIIS (Figure 1), and a 7-mm ball tip feeler was inserted into the channel to ensure its completion. Subsequently, 7.5-mm diameter iliac screws were placed. Finally, L4–L5 pedicle screws and iliac screws were connected by a curved rod, and a cross-link was fixed between the L5 pedicle screws and the iliac screws.

### Biomechanical tests

**Figure 2:**
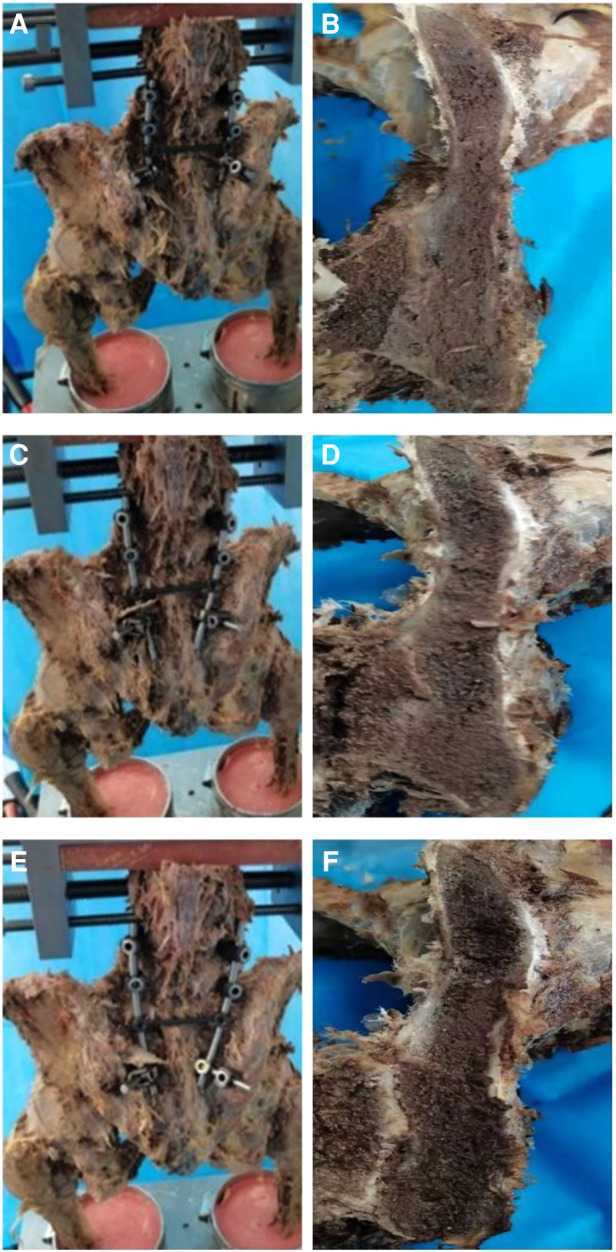
(**A**) Placed iliac screw from PSIS to AIIS. (**B**) Cross-sectional view of the channel from PSIS to AIIS. (**C**) Placed iliac screw from 1 cm medial and 1 cm caudal of PSIS to AIIS. (**D**) Cross-sectional view of the channel from 1 cm medial and 1 cm caudal of PSIS to AIIS. (**E**) Placed iliac screw from 2 cm below PSIS to AIIS. (**F**) Cross-sectional view of the channel from 2 cm below PSIS to AIIS.

The pelvic specimens were fixed on the special fixture of the American E10000 material mechanics testing machine (provided by the Institute of Orthopaedics, Soochow University) (Figures 1, 2). The L3 vertebral body was kept in a horizontal state during the experiments. The bilateral anterior superior iliac spine and pubic symphysis were placed in the same coronal plane to simulate the force on the pelvis when standing. The compression load of the L3 vertebral segment was 0–500 N (17), and the stress load speed was 3 mm/min through the upper loading connector. The analysis software Bluehill 2.0, provided by the mechanical testing machine, automatically recorded the load–displacement curve and calculated the compressive stiffness (N/mm). Three channels needed to be tested on each specimen, one channel was randomly selected for 3 consecutive measurements, and the next channel was also tested 3 times before proceeding until all three channels were measured and the results were averaged. After each test cycle was completed, the pelvic specimen was thoroughly inspected, and we observed that the internal fixators did not loosen or break. The next channel test was randomly carried out. In the torsional load experiment, a 6 N·m torsional load was applied to the specimen through the rotational axis. The torque-torsional angle curve was automatically recorded by WaveMatrix software, and the torsional stiffness (N·m/°) was calculated. Similarly, each specimen was tested three times, and the average rotation angle was calculated. Normal saline was sprayed regularly to keep the specimen moist during the experiment.

### Finite element injury models and finite element analyses

**Figure 3:**
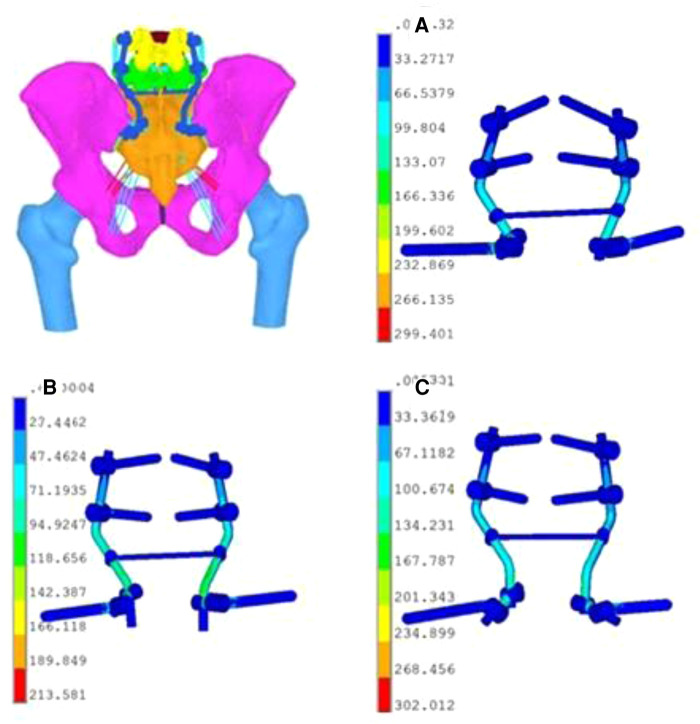
Established finite element model of lumbo-iliac fixation: under the vertical working condition. (**A**) Stress distribution nephogram of internal fixators in channel A. (**B**) Stress distribution nephogram of internal fixators in channel B. (**C**) Stress distribution nephogram of internal fixators in channel C.

One normal adult male volunteer (48 years old, 175 cm, 70 kg) was recruited. The study protocol was approved by the Ethics Committee of Shandong Provincial Hospital. The volunteer agreed with written informed consent. The radiographic data of the lumbar spine, pelvis, and femur were obtained by CT scan (Siemens Spiral CT, Germany, 0.625 mm slice thickness, 0.625 mm the interval and 512 * 512 the pixel) and imported into Mimics 21.0 (Materialise, Belgium) in Dicom format. Then, these files were processed by Geomagic Studio 12.0 (Geomagic, USA). Pro/Engineer 5.0 (PTC, USA) and Hypermesh 2017 (Altair, USA) were used to draw the iliac screw, pedicle screw, longitudinal rod, connector, transverse connecting rod, reconstruction plate, screw, etc. The material properties and characteristics, Young's modulus, and the structure of the model ligament were set. The three-dimensional finite element model of the L4 pelvic-proximal femur, pedicle screw, and iliac screw were imported into Ansys 19.0 (SASI, USA) for finite element analyses (Figure 3). The finite element model included the lumbar (L4 and L5), pelvis, and proximal femur. The full pelvis was composed of the left ilium, sacrum, right ilium, and pubic symphysis, and these bones consisted of cortical bone and cancellous bone. The anterior sacroiliac, interosseous sacroiliac, posterior sacroiliac, sacrotuberous, and sacrospinous ligaments were also created to simulate normal conditions. Between the plate-screw, screw-connecting rod was established as a whole, there was no universal movement. The binding constraint between screw and bone was established by coupling. Linear elastic isotropic material properties were used, and the properties of the bones and ligaments are shown in Table 2 (17–19). To validate the reasonableness of the model, the present model was compared with the experimental data of previous scholars (20–22). Model was subjected to 500 N concentrated force and 10 N·m moment, and its range of motion (ROM) was measured under four states of motion: flexion, extension, lateral bending, and axial rotation. It was concluded that the ROM of model was in good agreement with the results of other scholars' studies, which proved the validity of this experimental model. Similarly, the pelvic Tile C1 injury model (pubic symphysis separation, left sacral Denis II fracture) was established. Simultaneous 5-hole reconstruction plate fixation of the pubic symphysis and lumbo-iliac fixation for posterior pelvic ring surgery. Finite element analyses were used to explore the biomechanical characteristics of the bilateral single iliac screws, divided into three channels: channel A from PSIS to AIIS, channel B from 1 cm medial and 1 cm caudal of PSIS to AIIS, channel C from 2 cm below PSIS to AIIS. The number of elements for implants was 1,713,729 for channel A, 1,715,997 for channel B, and 1,713,492 for channel C. The number of nodes for implants was 2,794,487 for channel A, 2,798,784 for channel B, and 2,795,149 for channel C. The standing state of the human body was simulated, and the boundary conditions were set at the bilateral femoral ends. A 500 N vertical downwards load was applied to the upper surface of the L4 vertebral body, and torque in different directions of 10 N·m was applied to simulate the working conditions of flexion, extension, lateral bending, and rotation. Finite element software obtained the stress nephogram, displacement nephogram, and deformation nephogram of the internal fixation, vertebral body, and iliac bone. The maximum von Mises stress of internal fixation and the maximum von Mises stress of the vertebral body and ilium in different channels were then compared.

**Table 2:** The properties of material used in finite element model.

Material	Youngs modulus (MPa)	Poisson’s ratio *U*	*K* (N/mm)
Cortical bone (vertebral body)	12,000	0.3	
Cancellus bone (vertebral body)	345	0.2	
Posterior element (vertebral body)	3,500	0.3	
Cortical bone (ilium)	17,000	0.3	
Cancellus bone (ilium)	132	0.2	
Cortical bone (sacrum)	6,140	0.3	
Cancellus bone (sacrum)	1,400	0.3	
Symphysis pubis	5	0.45	
Articular cartilage	100	0.3	
Plates (titanium alloy)	110,000	0.3	
Screws (titanium alloy)	110,000	0.3	
Sacroiliac posterior long ligament			1,000
Sacroiliac posterior short ligament			400
Sacroiliac anterior ligament			700
Sacrotuberous ligament			1,500
Sacrospinous ligament			1,400

### Statistical methods

The maximal compressive displacement and torsional angle were obtained. The following formulas were used to calculate the compressive and torsional stiffness of the fixation construct:

Compressive stiffness = 500 (N)/maximum compressive displacement (mm)
Torsional stiffness = 6 (N·m)/maximum torsional angle (°)

SPSS software (version 20.0; Chicago, IL, USA) was used for the statistical description and analysis of the experimental results (*n* = 5). The measurement data are expressed as the mean ± standard deviation (SD). One-way analysis of variance was used for comparisons between groups. The least significant difference method was used for pairwise comparisons. The difference was statistically significant when *P* < 0.05.

## Results

### Biomechanical tests

After the unstable pelvic ring injury model was fixed with three channels of bilateral single iliac screws, the overall displacement of pelvic specimens under a 500 N vertical load was greater than that of complete pelvic specimens (1.852 ± 0.104 mm), and the difference was statistically significant (*P* < 0.05) (Table 3). The vertical displacement fixed by channel B (2.054 ± 0.248 mm) was smaller than that fixed by channel A and channel C (2.153 ± 0.175 mm and 2.370 ± 0.167 mm, respectively), and the difference in the vertical displacement fixed by channel B and channel A was not statistically significant (*P* > 0.05). The compressive stiffness of the bilateral single iliac screws in the three channels was significantly lower than that of the intact pelvic specimens (270.7 ± 15.38 N/mm), and the difference was statistically significant (*P* < 0.05) (Table 3). The compressive stiffness of channel B (246.15 ± 27.85 N/mm) was greater than that of channel A and channel C (233.43 ± 18.5 N/mm and 211.79 ± 14.58 N/mm, respectively), but there was no significant difference between channel B and channel A (*P* > 0.05). The compressive stiffness of channel A and channel C was significantly different from that of complete pelvic specimens (*P* < 0.05).

**Table 3:** Vertical displacement and compressive stiffness of pelvic specimens under 500 N (mean ± SD).

					*P* Value					
	Control Group	Channel A	Channel B	Channel C	Control Group vs. A	Control Group vs. B	Control Group vs. C	A vs. B	A vs. C	B vs. C
Displacement (mm)	1.852 ± 0.104	2.153 ± 0.175	2.054 ± 0.248	2.370 ± 0.167	0.011*	0.132	<0.01*	0.48	0.079	0.045*
compressive stiffness (N/mm)	270.7 ± 15.38	233.43 ± 18.5	246.15 ± 27.85	211.79 ± 14.58	0.009*	0.123	<0.01*	0.42	0.074	0.04*

* Indicated statistically significant.

The overall torsional angle of pelvic specimens fixed with three channels of bilateral single iliac screws under a 6 N·m torsional load was greater than that of the complete pelvic specimens (2.419 ± 0.176°), and the difference was statistically significant (*P* < 0.05) (Table 4). The torsion angle of pelvic specimens fixed by channel B (2.708 ± 0.280°) was less than that of pelvic specimens fixed by channel A and channel C (2.973 ± 0.274° and 3.411 ± 0.197°, respectively), and there was no significant difference between channel A and channel B. The torsional stiffness of the bilateral single iliac screws with three channels was significantly lower than that of the complete pelvic specimen (2.491 ± 0.184 N·m/°) (*P* < 0.05). The torsional stiffness of channel C (1.764 ± 0.101 N·m/°) was smaller than that of channel A and channel B (*P* < 0.05) (Table 4). There was no significant difference in torsional stiffness between channel A (2.032 ± 0.187 N·m/°) and channel B (2.234 ± 0.223 N·m/°) (*P* > 0.05).

**Table 4:** Torsional angle and torsional stiffness of specimens under a torsional load of 6 N·m (mean ± SD).

					*P* Value					
	Control Group	Channel A	Channel B	Channel C	Control Group vs. A	Control Group vs. B	Control Group vs. C	A vs. B	A vs. C	B vs. C
Torsional angle (°)	2.419 ± 0.176	2.973 ± 0.274	2.708 ± 0.280	3.411 ± 0.197	0.005*	0.086	<0.001*	0.169	0.019*	0.001*
Torsional stiffness (*N*·m/°)	2.491 ± 0.184	2.032 ± 0.187	2.234 ± 0.223	1.764 ± 0.101	0.004*	0.081	<0.001*	0.159	0.022*	0.003*

* Indicated statistically significant.

### Finite element analyses

**Figure 4:**
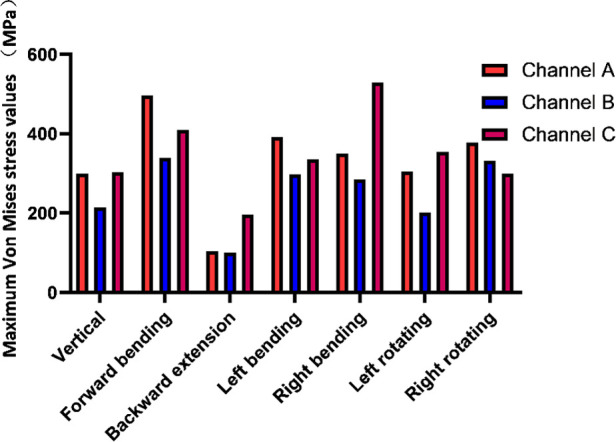
Maximum von mises stress of internal fixation with three channels.

In terms of the overall stress distribution nephogram, the maximum von Mises stress was shown on the internal fixator. There was a large stress concentration in the iliac screw, connector and longitudinal rod, so that the tail of the iliac screw and the surroundings of the connector were more pronounced (Figure 3). The maximum von Mises stress of the internal fixator fixed in channel B under the working conditions of vertical, forwards bending, backwards extension, left bending, left rotation and right bending (213.98 MPa, 338.96 MPa, 100.63 MPa, 297.06 MPa, 200.95 MPa, 284.75 MPa, respectively) were significantly lower than that fixed in channel A and channel C (Figure 4, Table 5). Similarly, under various working conditions, the maximum von Mises stress of the internal fixator of channel B was less than that of channel A (Figure 4). Under the vertical condition, the maximum von Mises stress of the internal fixator in channel B was 71.4% of that in channel A. Under the left rotation condition, the maximum von Mises stress of the fixator channel B was only 65.9% of that in channel A. This showed that the maximum von Mises stress of the fixator in channel B was the smallest, the fatigue resistance was strong, and the fixator was less prone to broken screws.

**Table 5:** Maximum von mises stress of internal fixation with three channels (MPa).

	Channel A	Channel B	Channel C
Vertical	299.4	213.98	302.01
Forward bending	496.16	338.96	408.96
Backward extension	103.05	100.63	195.73
Left bending	390.56	297.06	335.09
Right bending	349.19	284.75	528.56
Left rotating	304.91	200.95	354.27
Right rotating	377.97	331.35	298.72

Under the working conditions, except extension, the maximum von Mises stress of the vertebral body was as follows: channel A < channel B < channel C. In terms of the maximum von Mises stress of the iliac, under the conditions of upright, forwards bending, and left bending and right rotation, channel A maximum was > channel B. The results showed that the overall stress distribution of channel B was more reasonable.

## Discussion

Spinal-pelvic fixation can treat many complications of trauma surgery and spinal surgery (23) by aiming to rebuild the stability of the spine and pelvis in cases such as traumatic spinal pelvic separation (“H or U”-shaped fractures of the sacrum, etc.), complex sacral comminution fractures, sacrum fracture complicated with nerve injury, spinal protrusion deformity, sacrum tumour, tuberculations of the lumbar and sacrum, severe lumbar spondylolysis, and scoliosis combined with pelvic tilt. Lumbo-iliac fixation for treating U-type sacral fractures has been reported in many studies, and this treatment can provide multiplanar stability (24, 25). Fujibayashi et al. (26) used iliolumbar fixation for palliative treatment of destructive sacral tumours and achieved satisfactory clinical efficacy. The authors pointed out that this fixation method could increase the stability of the lumbosacral region. Ebata et al. (27) applied the spinal-pelvic fixation system in adult spinal deformity correction and achieved satisfactory results. By finite element analysis, Song et al. (19) compared the biomechanical characteristics between bilateral and unilateral lumbo-iliac fixation in unilateral comminuted sacral fractures. The results revealed that the stability of unilateral lumbo-iliac fixation was insufficient to reconstruct the posterior pelvic ring. Furthermore, unilateral fixation may lead to an imbalance of the lumbar vertebra and pelvis. In contrast, bilateral lumbo-iliac fixation could provide satisfactory stability and lumbar balance. Therefore, bilateral lumbo-iliac fixation was selected in this study, and the finite element results showed that the stress distribution nephograms of L4 and L5 and the acetabulum and femur were balanced. Because the fracture line was on the sacrum, the stress distribution in the sacrum was uneven. The results were consistent with those of Song et al. (19).

Certainly, if we opt for incisional repositioning of the bilateral lumbo-iliac fixation, then there are problems to keep in mind, such as a longer operating time, higher amounts of bleeding and surgical trauma. These problems are also independent risk factors for the occurrence of postoperative infection. In some cases, when we specify the optimal channel for placement of the iliac screw in lumbo-iliac fixation, we can use a smaller incision and minimally invasive techniques to treat the fractures. Futamura et al. (28), Koshimune et al. (29), and Okuda et al. (30) used small incisions and minimally invasive lumbo-iliac fixation to reduce the risk of skin and soft tissue injury and infection and improve the healing rate of fractures.

In lumbo-iliac fixation, L4–L5 fixation was performed with pedicle screws in this study. Long fusion offers better mechanical stability and bone fusion. However, this inhibits the patient's range of motion during the postoperative period. Schildhauer et al. (31) and Shen et al. (32) suggested fusion from L4 to the pelvis. Concerning patients after total sacrectomy, Zhang et al. (33) recommended reconstruction from L3 to the pelvis. In sacral tumour patients, Acharya et al. (34) executed L4-iliac fusion and screw-rod instrumentation using the bilateral dual iliac screw technique. All these reports indicated that patients could achieve a good result after reconstruction.

The iliac screw technique in spine-pelvis fixation has been widely used, and reliable lumbar and pelvis stability has been obtained clinically (9). There are many options for iliac screw placement sites and channels. The iliac screw technique theoretically fixes the length behind the iliac bone to the maximum extent so that the strength of the screw fixation is the strongest, and the prominence of the internal fixation is avoided. At present, it is not clear which channel of the iliac screw is best for fixation. In addition, Miller et al. (35) studied the anatomy of cadaver pelvic specimens. They believed that when the length of the inserted iliac screw located in the channel from PSIS to AIIS reached 100 mm, there was a 25% probability of penetrating the acetabulum and entering the joint. Therefore, they suggested that the length of the iliac screw should be less than 90 mm. Moshirfar et al. (36) performed a retrospective analysis of the literature on the clinical application of iliac screws. They believed that for high effectiveness and safety, the length of iliac screws should be greater than 80 mm, and the diameter should be 7.5 mm. Therefore, the use of iliac screw lengths ranging from 70 mm (above the level of the greater sciatic notch) to 90 mm has become a clinical consensus. In this study, the iliac screw was 80 mm in length and 7.5 mm in diameter, and all iliac screws were within the iliac channel and did not exceed the total length or diameter of the channel.

This study determined the biomechanical differences of three channels of bilateral single iliac screws in the inferior iliac column. Compared with previous biomechanical studies, such as sacroiliac screws, posterior ring tension band plates and sacrum rods (37, 38), this study showed that three channels of bilateral single iliac screws in lumbo-iliac fixation could effectively restore the stability of the reconstructed pelvis. The compressive stiffness of channel A was 86.2% of that of the complete pelvis, the compressive stiffness of channel B was 90.9% of that of the complete pelvis, and the compressive stiffness of channel C was 78.2% of that of the complete pelvis. The biomechanical experimental method of cadaveric specimens could clearly show the displacement of the pelvic specimen model and the structural rigidity of the internal fixator after fixation. However, the experimental results were susceptible to many factors, such as specimen quality, specimen source, specimen difference, theoretical knowledge of the tester, and the operation level of the tester, etc. The choice of specimen damage model has a great influence on biomechanics. Zheng et al. (14) studied the effect of iliac screw insertion depth on the stability and strength of lumbo-iliac fixation constructs and concluded that after total sacrectomy, the lumbo-pelvic reconstruction using short and long iliac screws restored 53.3% and 57.6% of the initial stiffness in compression testing, respectively. Yu et al. (13) studied that in compression, the stiffness of the L3-iliac fixation constructs of Single-Short, Single-Long were 73% and 76% of the intact state, respectively, and more segments of the lumbar spine were selected for fixation. Wu et al. (17) performed biomechanical studies of three kinds of internal fixation for the treatment of sacroiliac joint disruption using biomechanical test and finite element analysis, and under a vertical load of 500 N, the average displacements of the pelvis fixed with an anterior plate were the largest one (4.704 ± 0.600 mm). The value of the pelvis fixed with two sacroiliac screws was in the middle (3.128 ± 0.519).

According to the pelvic specimens' compressive and torsional stiffness, there was no significant difference between channel B and channel A. Still, the results of channel B fixation were greater than those of channel A fixation. We believe this is due to the fixed distance between channel A and channel B being incredibly close, with both of them being close to the standard mechanical conduction path. The fixed strength of channel B is better than that of channel A. The study also showed that the construct stiffness of channel C was the worst. The fixed position of channel C is lower. When the pelvic specimen is in a standing position, the normal force conduction path is higher than the fixed position of channel C, resulting in a sizable vertical displacement of the pelvis and a weakened fixation strength. When the human body is sitting, the standard mechanical conduction path moves down, making the fixation of channel C close to normal and having a high fixation strength. However, we did not perform a sitting-time biomechanical test, and this interpretation requires further confirmation.

In this study, specimens stored in a 10% formalin solution for two weeks were used. The use of formalin-fixed bones for biomechanical investigations is controversial, because formalin fixation is thought to change the biomechanical properties of bone. No evidence for changes in bone density (39), bone mineral density (40), crystallinity of the bone apatite (41) or the histological structure and quality of soft tissues (42, 43) have been observed as a result of formalin fixation. However, aldehydes (e.g., formaldehyde) affect a significant number of inter- and intrafibrillar crosslinks in collagen molecules (44) and therefore can influence the biomechanical behaviour of bone. A small number of biomechanical studies focusing on the effects of formalin fixation on the biomechanical properties of animal bone have been published. Studies using human bones are rare. Moreover, these studies are hard to compare because of different fixation methods, fixation duration and different test set-ups. Currey et al. (45) found that fixing bovine bone in a 10% formalin solution for 3 h slightly but significantly increased the bending Young's modulus (+2%) and largely decreased the impact energy (−46%). Goh et al. (46) performed torsion tests on cat humeri and four-point bending tests on cat femora. Specimens were stored in a 10% formalin solution for 3 or 21 days. It was found that the formalin preservation did not alter the ultimate load and stiffness, but largely reduced the energy absorption capacity. Furthermore, no significant differences were found between the specimens fixed for 3 and 21 days. Sedlin and Hirsch (47) tested cortical specimens from a human femur in tension. The specimens were elastically tested, then placed in a 10% formalin solution for 3 weeks and thereafter retested. The Young's modulus was determined and no significant difference was found between the two testing occasions. Burkhart et al. (48) compare axial and torsional stiffness and bone mineral density in fresh and embalmed human bones (preserved in 4% formalin solution for 6 weeks). The formalin group showed significant higher stiffness values for torsional and axial loads than the fresh group. These differences were not reflected in bone mineral density values. Therefore, we considered the results of the selected specimens to be credible.

This study simulates the vertical displacement of a specimen in a normal human body in a bipedal stance with a vertical load of 500 N. The vertical load of 500 N is approximately the weight of the upper body (17). A systematic review of the literature found that there was no homogeneous criterion for the magnitude of torsional load. Some researchers used 7.5 Nm (49) and 8 Nm (14), while others applied torsional moments from 0 to 5, 10, 15, and 20 Nm (50). We chose 6 N·m torsional load within a reasonable range. This justified is *via* biomechanics, so 500 N compression load and 6 N·m torsional load can be used for biomechanical studies of pelvic stability in the experiment work. And the experimental results are also plausible. In addition, due to the small number of cadaver specimens, individual differences, and multiple biomechanical tests performed by multiple internal fixation methods in the same specimen, etc, these factors affect the results of biomechanical experiments. And the use of cadaver specimens for biomechanical experiments, the specimen consumption damage. The greater the torque, the greater the damage to the specimen.

Finite element analysis has been widely used in the field of medical research. It is primarily based on the digital model of evaluating the immediate stability of the body's internal fixation stability (overall stability and local stability), the body's stress distribution nephogram and rigid structure, the maximum von Mises stress and stress distribution nephogram of internal fixation and the stress distribution of the adjacent structures (51). Comprehensive and accurate results can be obtained without huge cost and repeatable experiments without wasting injury on samples. Besides, stress changes and stress distribution of internal fixation instruments before and after surgical fixation can also be predicted and analyzed. It has the advantage that traditional cadaver specimen biomechanical experiment does not have. Yamamoto et al. (20) studied three-dimensional movements of the whole lumbar spine and lumbosacral joint. From preliminary experiments, 10 N·m was judged to be sufficient to produce maximum physiologic motions, but small enough not to injury the specimen. Later, many scholars studied torque at 10 N·m (20–22). Therefore, we chose a torque of 10 N·m in the finite element analysis.

In this study, the results showed that the maximum von Mises stress of the model shown on the internal fixation was the largest, followed by the vertebral body, while the maximum von Mises stress of the iliac bone was the smallest. The stress of the internal fixation device should not be excessively concentrated at a certain point in order to avoid a fracture of the screw rod system due to excessive stress concentration (37). In addition, a standard reflecting the safety performance of the internal fixation device is the maximum stress it bears. After applying the load, the greater the maximum stress of the internal fixation is, the greater the possibility of complications such as broken screws and broken rods and the failure of the fixation device. This study showed that the iliac screw, connector, and longitudinal rod had high stress concentrations. The stress nephogram of the internal fixation showed that the maximum von Mises stress was mainly concentrated on the tail of the iliac screw and around the connector, which was the position where the screw-rod system was prone to fracture in the spine-pelvic fixations. In addition, the maximum von Mises stress of internal fixation in channel B under all the conditions was smaller than that in channel A. The maximum stress value of internal fixation in the C channel was the largest of the three channels. The results showed that the stress distribution of channel B was scattered, the maximum von Mises stress of the internal fixation was small, and the fatigue resistance was strong.

An important criterion for evaluating surgical quality is the stress on the intervertebral disc after the operation. The greater the maximum stress on the intervertebral disc is, the more likely it will lead to degenerative changes of the intervertebral disc and symptoms such as low back pain (52). In all conditions except extension, the maximum von Mises stress of the vertebral body: channel A fixation was less than channel B fixation, and channel B fixation was less than channel C fixation. The maximum stress of the iliac bone fixed by channel A was greater than that of the iliac bone fixed by channel B under the conditions of upright, flexion, left bending and right rotation. The results showed that the overall stress distribution of the channel B fixation model appeared to be more reasonable.

There were some defects in this study. First, cadaver specimens were used for biomechanics, the number of specimens was small, and there were differences among specimens. Multiple fixation devices have been tested on the same specimen. Still, the influence between the fixation devices before and after fixation could not be eliminated, and errors could not be avoided. To avoid specimen destruction from numerous testing steps, 3 cycles of physiologic compression and torsion loading were performed and recorded for each type of channel fixation. At the same time, we analysed the recorded data, and if there was too much difference between the data, we discarded the unreasonable data and conducted the test again. The use of fresh, frozen cadaver specimens can provide first-hand clinical information. However, due to the limitation of sample size, source of cadaver specimens, and research funding, we were unable to use them in this study. We will use fresh, frozen cadaveric specimens in future studies to further validate our conclusions. In addition, the limited conditions in our orthopedic laboratory and the lack of specialized radiological equipment made it impossible to assess preoperative and postoperative pelvic fracture displacement from radiographic images (x-ray) at that time. At the end of the biomechanical experiments, the specimens were osteotomized to observe the integrity of each channel. Second, the establishment of the finite element model involved many aspects, and there was a disparity between the constructed pelvic model and the cadaveric pelvises. The assignment of bone material properties is a complex problem to solve, and at present, the characteristics of bone heterogeneity and anisotropy cannot be well simulated. At the same time, this study did not simulate the muscle structure around the human pelvis, which limited the freedom of the femoral tip in six directions and could not simulate the normal physiological activity of the human body. Third, the biomechanical experiment only verifies the biomechanical properties of a specific fixation method, which can only be used as a reference for clinical application. We should also consider the short-term and long-term clinical effects of the procedures.

## Conclusions

Biomechanical tests of pelvic specimens treated with a lumbo-iliac fixation for unstable posterior pelvic ring injury showed that bilateral single iliac screws with three channels could effectively restore the stability of the reconstructed pelvis. The compressive stiffness and torsional stiffness of channel B fixation were better than those of channel A fixation. The compressive stiffness and torsional stiffness of channel C fixation were the worst. Finite element analyses showed that channel B fixation has excellent biomechanical stability, a more reasonable overall stress distribution, a smaller internal fixation maximum stress value, a stronger fatigue resistance, and more resilience to the breakage of nails. These results suggest that for clinical application of lumbo-iliac fixation, the optimal iliac screw channel is 1 cm medial and 1 cm caudal of the posterior superior iliac spine to the anterior inferior iliac spine.

## Data Availability

The original contributions presented in the study are included in the article/Supplementary Material, further inquiries can be directed to the corresponding author/s.
